# Overview of Platelet Physiology: Its Hemostatic and Nonhemostatic Role in Disease Pathogenesis

**DOI:** 10.1155/2014/781857

**Published:** 2014-03-03

**Authors:** Kakali Ghoshal, Maitree Bhattacharyya

**Affiliations:** Department of Biochemistry, University of Calcutta, 35 Ballygunge Circular Road, Kolkata 700019, India

## Abstract

Platelets are small anucleate cell fragments that circulate in blood playing crucial role in managing vascular integrity and regulating hemostasis. Platelets are also involved in the fundamental biological process of chronic inflammation associated with disease pathology. Platelet indices like mean platelets volume (MPV), platelets distributed width (PDW), and platelet crit (PCT) are useful as cheap noninvasive biomarkers for assessing the diseased states. Dynamic platelets bear distinct morphology, where **α** and dense granule are actively involved in secretion of molecules like GPIIb , IIIa, fibrinogen, vWf, catecholamines, serotonin, calcium, ATP, ADP, and so forth, which are involved in aggregation. Differential expressions of surface receptors like CD36, CD41, CD61 and so forth have also been quantitated in several diseases. Platelet clinical research faces challenges due to the vulnerable nature of platelet structure functions and lack of accurate assay techniques. But recent advancement in flow cytometry inputs huge progress in the field of platelets study. Platelets activation and dysfunction have been implicated in diabetes, renal diseases, tumorigenesis, Alzheimer's, and CVD. In conclusion, this paper elucidates that platelets are not that innocent as they keep showing and thus numerous novel platelet biomarkers are upcoming very soon in the field of clinical research which can be important for predicting and diagnosing disease state.

## 1. Introduction

Platelets were discovered by Giulio Bizzozero in 1882 [1], but for many decades the dynamic and multifunctional nature of platelets remained a field of interest only for biologists. Anucleate, discoid platelets are the smallest blood particles which unveil their dynamicity through their morphology. Primarily they are associated with hemostasis, which is to initiate blood coagulation. Although very dynamic, they usually prefer to remain in inactive state and get activated only when a blood vessel is damaged. But hemostasis or blood coagulation is not the sole function of platelets; rather it is employed in several multifunctional attributes monitoring the homeostasis of the body. Its high sensitivity to different disease states eventually assigned it to be one of the most accessible markers. While keeping interactions with leukocytes and endothelial cells, it restores its behaviour as an important inflammatory marker [2]. Platelet reactivity for different disease pathogenesis is widely dependent upon some biologically active markers like CD36, CD41, CD42a, CD42b, and CD61. These include some active surface receptors and platelet secretory products. Platelet tends to alter the expression and signaling of these markers in different disease diagnosis and prognosis, providing a huge field to explore disease progression.

Primarily, platelet activity is associated with the initiation of coagulation cascades. Damage in blood vessel makes the subendothelial surface the primary target site of platelet action, where it establishes the hemostasis. Various proaggregatory stimuli also known as platelet agonists promote the action of platelet adhesion to the subendothelial surfaces. During this process, platelet changes its shape, releases its granule contents, and gradually forms aggregates by adhering with each other [3]. Thus its primary activity remains associated with minimizing blood loss. However, as discussed earlier platelets are not only confined in regulating hemostasis and thrombosis, but they also play many pivotal roles in disease pathophysiology. Platelet interaction and cardiovascular disease progression remain an unsolved riddle for many years [4]. Platelet hyperaggregation among the diabetic patients with CVD remains another striking area to explore. Platelet hyperactivity in various diseases provokes adverse effects in some cases, especially in coronary artery disease where hyperaggregation obstructs blood circulation.

Expression of platelet markers can be well studied by ELISA or Western blot. However, till date flow cytometry is the best standardized method to study platelet function [5, 6]. In this paper, we have tried to elucidate various aspects of platelets structure and function and their potential role in disease pathophysiology.

## 2. Versatility of Platelets: Its Structural and Functional Aspects

### 2.1. Ultrastructure

Platelet ultrastructure reveals its behavioural peculiarities. Megakaryocytes of the bone marrow are site of platelet formation. Diameter of a mature platelet is 2-3 *μ*m, which usually remains alive for 5–9 days. Approximately 2/3 of the platelets circulate in the blood and 1/3 is stored in the spleen. The normal platelet count is (150–400) × 10^3^ per microliter of blood. Each megakaryocyte can produce 5000–10000 platelets. An average healthy adult can produce 10^11^ platelets per day; old platelets are destroyed by phagocytosis in the spleen and liver (Kupffer cells).

**Figure 1:**
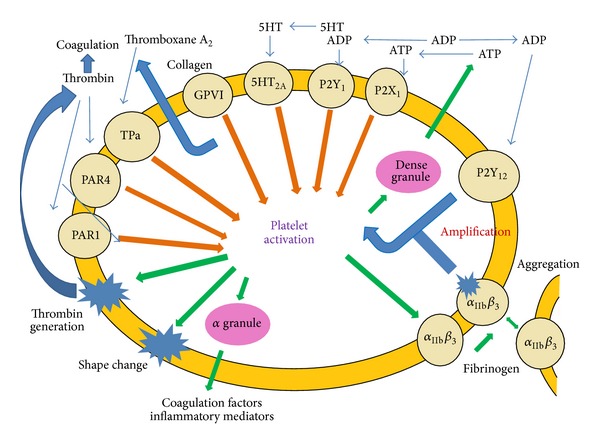
Platelet-activation mechanisms and role of the P2Y_12_ receptor. Platelet activation leads to dense-granule secretion of ADP, which activates P2Y_12_, inducing amplification of aggregation, procoagulant, and proinflammatory responses (adapted from Storey, 2008 [7]).

Platelets are unique in their structural assembly, though they are anucleate but have distinct mitochondria. Platelet plasma membrane, composed of phospholipid bilayer, is the site of expression of various surface receptors and lipid rafts which helps in signalling and intracellular trafficking. These markers include CD36, CD63, CD9, GPCR, IIbIIIa, and GLUT-3. These surface receptors also trigger the release of *α* granules which play a role in multiple functions, namely, coagulation, inflammation, atherosclerosis, antimicrobial host defense, angiogenesis, wound repair, and tumorigenesis [9]. Among these surface receptors, GPCR has been reported to play crucial role in ADP secretion from dense granules which is its major secretory product [10]. Asymmetrically arranged phospholipids (e.g., phosphatidylserine and phosphatidylinositol) present in the inner layer of the plasma membrane maintain the stability of its surface during nonprocoagulant state. During activation (Figure 1) platelet surface gradually exposes aminophospholipids by ATP-dependent floppases and scramblases to initiate coagulation cascades [11]. The open canalicular system (OCS) is the “tunnel” system present throughout the platelet cell and remains connected with the plasma membrane [12]. The major role of OCS is to give entry of external elements into the platelets as well as to release its granule contents to the exterior. Other than being a major storage site for plasma membrane glycoproteins, it facilitates the formation of filopodia during platelet activation [13]. Dense tubular system of platelets is a closed-channel network of residual endoplasmic reticulum and primarily involved in calcium sequestration with the help of cascades of reactions involving the activation of G protein-coupled receptor PAR-1 [14, 15]. The highly specialized cytoskeleton of platelets maintains its discoid structures as well as protects the cell from getting sheared in bloodstream. It has three major components: (1) the spectrin-based membrane skeleton, (2) the actin cytoskeleton, and (3) the marginal microtubule coil.

Platelets have two major storage granules, namely, *α* and dense granules, whose function is to store biologically active molecules precisely involved in initiation of coagulation and recruiting other cells during inflammation [16]. The more prevalent *α* granule contains proteins (e.g., GPIIbIIIa, fibrinogen, and vWf) which initiate the coagulation cascades. Numerous membrane proteins essential to platelet function are also packaged into *α* granule which includes GPIIbIIIa, P-selectin (CD62P), and CD36. *α* granules also have the bulk of cellular P-selectin in their membrane. P-selectin via P-selectin glycoprotein ligand (PSGL1) has been reported to recruit neutrophils [17, 18]. Dense granules store a variety of hemostatically active molecules which are secreted during platelet activation; these include catecholamines, serotonin, calcium, adenosine 5′-diphosphate (ADP), and adenosine 5′-triphosphate (ATP). ADP is a weak platelet agonist, triggering platelet shape change, granule release, and aggregation [19].

### 2.2. Platelet Receptors

Platelet surface receptors have been always a field of interest among scientists for many years and platelets also can exert their granule contents during disease prognosis. A list of platelet receptors, also known as platelet agonists, have been summarized in (Table 1) according to their activity [20].

### 2.3. Platelet Endothelium Interaction, Hemostasis, and Platelet Aggregation

Platelets are completely different from endothelial cells and can interact in multiple ways when exposed to endothelial surface (Table 2). These interactions can be of cross talk over a distance also known as paracrine signaling via transient interactions or through receptor mediated cell- cell adhesion. Platelets are also able to release or transfer many substances as discussed earlier that also interact with endothelial cell [21]. Although platelets and endothelial cells are different in many ways, they do share some common features, like both cell types are derived from a common bone marrow derived progenitor cell. Some of their transcriptional networks and gene expression programs are also similar like GATA-2, vWf, multimerin, and P-selectin. Both of them store their bioactive materials in their cytoplasmic granules. From an evolutionary approach, endothelium can be considered as sedentary in its way where platelets and megakaryocytes circulate in bloodstream [22].

Endothelial cells with the help of COX-1, COX-2, and prostacyclin synthase can convert arachidonic acid into prostacyclin, which in turn inhibits platelet function by the elevation of intracellular cyclic AMP levels [23].

An injury in the vessel wall activates platelets to initiate coagulation, which is also known as hemostasis. Dynamic platelets readily get activated/inhibited by several endogenous and exogenous stimuli. They initiate primary hemostasis by adhering themselves to the damaged vessel wall. GPIb-V-IX and GPIa-IIa receptors and subendothelial compounds like vWf and collagen interact with each other to mediate this procedure (Figure 6). Binding of ligands to the GP receptors changes platelet shape as well as triggers the release of its granule contents, which ultimately leads to the formation of aggregates which are also known as “platelet plugs” or “white thrombi” (Figure 5).

Platelet starts to change its shape by the formation of pseudopods when intracellular Ca^2+^ concentration exceeds a specific threshold. During shape change, platelet fibrinogen receptors (GPIIb/IIIa) are exposed and activated, and platelet-platelet aggregation is initiated. This is also known as primary aggregation which is reversible. However, resting platelets are not able to bind fibrinogen. Arachidonic acid thromboxane pathway is an important platelet activation pathway (Figure 4). Aspirin, also known as acetylsalicylic acid, a drug widely used in CVD, inhibits platelet aggregation through irreversible acetylation and inactivation of COX, resulting in blockage of TxA_2_ production [24, 25]. Mature normal human platelets express only COX-1, as the anucleate platelet cannot synthesize enzyme *de novo*. As a result the effect of aspirin on them is permanent and cumulative. Thus, the cardioprotective effect of aspirin is exerted through the irreversible and permanent impairment of thromboxane A_2_-dependent platelet function, which reduces the development of acute arterial thrombosis [26].

ADP is another important platelet activator. P2Y_12_, an ADP specific receptor, is present on the platelet membrane and is coupled to inhibitory G-proteins and mediates ADP-induced release of Ca^2+^, inhibiting adenylate cyclase and activating the GPIIb/IIIa receptor which leads to platelet aggregation. The thienopyridines, ticlopidine, and clopidogrel inhibit platelet activation via blockage of the P2Y_12_ receptor [27]. Thromboxane A_2_, ADP, and other substances such as serotonin, released from the activated platelet, provide important positive feedback and strengthen the platelet-rich clot initiating secondary aggregation which is irreversible (Figure 3) [28].

Platelet response is amplified via substances released by platelet granules which recruit other platelets and blood cells. The platelet plug initially formed in primary hemostasis is relatively unstable. Coagulation cascade and formation of thrombin and fibrin prolong secondary hemostasis. During platelet activation, platelet membrane phospholipids become negatively charged, which facilitates coagulation activation (e.g., FV, FVIIIa, FIXa, and FX). Binding of the prothrombinase complex (FXa, FVa, Ca^2+^, and prothrombin) to the platelet membrane occurs in this step. Further platelet activation is initiated by the formation of thrombin. These cascades lead to the formation of “red thrombus” strengthening the blood clot [29].

Intact vascular endothelium releases two major antiaggregants, prostacyclin (PGI_2_) and nitric oxide (NO), which prevent the formation of thrombus inside the blood vessel [31].

### 2.4. Platelet Function Assay

Platelets are dynamic blood particles which can interact with each other as well as with leukocyte and endothelial cells. Previously, platelet function was assessed using light transmission aggregometer [32] whose primary function was to measure the increase in light transmission through a platelet suspension when platelets were aggregated by an agonist. It has some major drawbacks. First of all, the result obtained may be affected by many variables. Secondly, the accuracy and the reproducibility of the technique are very poor. And overall in case of low platelet count the result is very difficult to interpret. Thus this technique was almost replaced by flow cytometry assay [5, 6], which is far more convenient and perfect. Expression of several platelet markers such as CD62p (P-selectin) and CD63 is now being well studied using flow cytometry technique [33, 34]. PAC 1, a monoclonal (Figure 7) antibody with specific binding to the fibrinogen receptor of activated platelets, is now widely used to assess platelet activation. Platelet-bound antibodies were detected either by fluorescein isothiocyanate (FITC) or by phycoerythrin (PE) [35, 36].

As platelets are devoid of any nucleus, proteomics aids new therapeutic targets of many diseases with platelet dysfunction [37]. After obtaining platelet-rich plasma, platelets are isolated and simultaneously disrupted to analyze their proteomic contents. 1D or 2D gel electrophoresis is involved to separate the protein fractions where mass spectrometry can analyze the proteins. Further investigation involves the usage of Western blotting which will ultimately validate the obtained data. A researcher group found alteration in cytoskeleton as well as significant differences in ILK, GAPDH and PK in platelets from arterial thrombosis [38]. Another group found significant alteration in cytoskeleton, differential expression of Coronin-1B, PSB8, and pleckstrin, and upregulation of proteins associated with energy metabolism. Thus, proteomics assay could implicate a new horizon in platelet study [39].

### 2.5. Symptoms of Platelet Dysfunction and Clinical Assessment

Diagnosis of platelet function relies on clinical findings of both detailed medical history and family history. Some platelet disorders are hereditary where most of them are acquired due to various diseased conditions. An alteration in platelet function includes prolonged or excessive bleeding which is the primary screening procedure to assess platelet dysfunction. Change in platelet number and mean platelet volume are another indicating markers in platelet dysfunction. Some of the common symptoms include [40] the following:

- unexplained or extensive bruising particularly associated with soft tissue hematoma,
- epistaxis, particularly lasting more than 30 minutes or causing anemia,
- menorrhagia, particularly if present since menarche,
- gingival bleeding,
- heavy and prolonged bleeding childbirth,
- bleeding following invasive procedures (e.g., dental extraction, tonsillectomy, and adenoidectomy).

Clinically, platelet function is assessed either by checking platelet aggregation by taking the whole blood or platelet-rich plasma from the patients to the pathology lab or by point-of-care (POC) platelet function test. The tests are summarized in (Table 3).

## 3. Platelet Dysfunction in Disease Pathophysiology

In recent times, platelets have emerged to be important markers for disease pathophysiology. They are multifunctional blood particles and very important clinical targets for many disease pathophysiology (Figure 8). Being important inflammatory markers, they play important roles in atherosclerosis and cardiovascular disorders which are correlated with type 2 diabetes. They have profound role in tumor biology as well as allergic inflammation. Thrombin, an agonist released by platelets has profound role in inflammation [41–44], angiogenesis [45] and embryonic development [46, 47].

## 4. Platelet Dysfunction in Cardiovascular Disorder (CVD) and Diabetes

Diabetes mellitus is heterogeneous, multifactorial, polygenic disease characterized by defect in insulin's secretion (the beta cell secretory defect) and action (insulin resistance) [48]. Type 2 which is the most prevalent form is basically a lifestyle disorder now becoming a major global threat. Obesity is the major cause of diabetes in the adults [49]. The most prevalent diabetic macrovascular complication is cardiovascular disorder [50]. BMI was significantly and linearly associated with systolic blood pressure, fasting glucose levels, plasma total cholesterol, VLDL cholesterol, and LDL cholesterol levels and was inversely and linearly associated with HDL cholesterol level [51], having a direct correlation in developing T2DM. Obesity is a key feature of metabolic syndrome, reflecting the fact that the syndrome's prevalence is driven by the strong relationship between body mass index (BMI) and increasing adiposity [52].

Our research group previously found hyperglycemia-induced oxidative stress in structural functional alterations of hemoglobin and red blood cells [53, 54]. But there are limited reports to elucidate the altered behavior of platelets in different diseases, majorly focusing on diabetes and associated cardiovascular disorders which are major threats to society in recent times.

### 4.1. Hyperaggregation in Platelets

**Figure 2:**
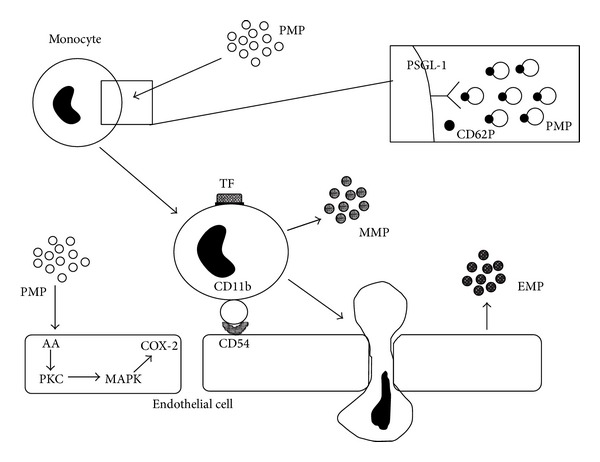
Mechanism of vascular changes by platelet-derived microparticles (PMPs). PMPs activate monocytes by a reaction between P-selectin and PSGL-1 (P-selectin glycoprotein ligand-1). Activated monocytes induce expression on the cell surface of tissue factor (TF) and CD11b. Activated monocytes also induce release of monocyte-derived microparticles (MMPs). PMPs induce COX-2 production in endothelial cells. PMPs enhance expression of CD54 (ICAM-1) on the endothelial surface. Activated endothelial cells also induce release of endothelial cell-derived microparticles (EMPs), enhancing adhesion between endothelial cells and monocytes. Finally, monocytes induce migration of endothelial cells, resulting in vascular changes. Abbreviations: arachidonic acid (AA); protein kinase C (PKC); mitogen-activated protein kinase (MAPK) (adapted from Nomura, 2001 [8]).

**Figure 3:**
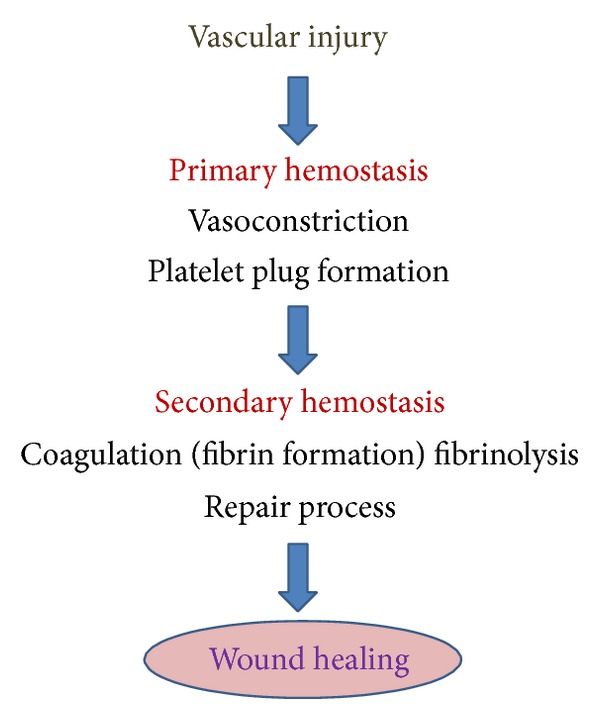
Pathway illustrating hemostasis.

**Figure 4:**
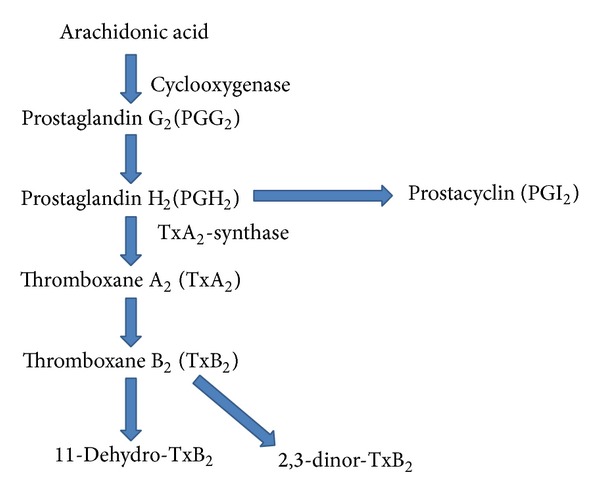
Thromboxane biosynthesis pathway.

**Figure 5:**
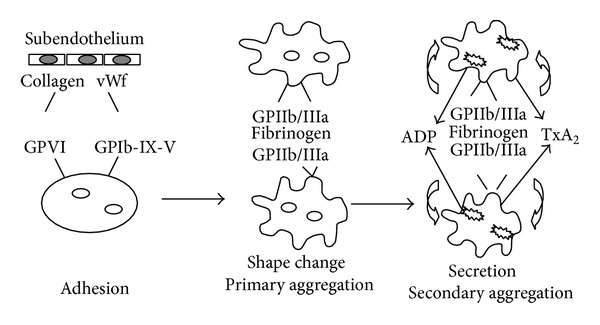
After an injury in the vessel wall, activation of platelets begins to start. It involves its adhesion to the subendothelium surface. Interaction between receptors like GPIb-V-IX, GPIa-IIa, and subendothelial compounds like vWf and collagen triggers the release of platelet granule contents accelerating aggregate formation.

**Figure 6:**
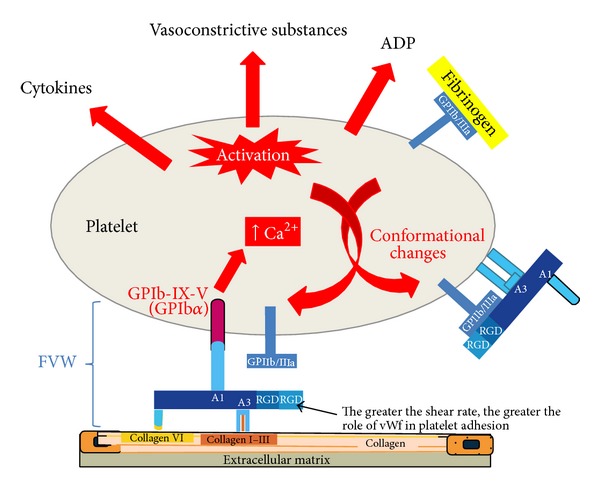
Diagram illustrating the role of von Willebrand factor (vWf) in platelet adhesion. High flow rates induce conformational changes in vWf allowing interaction of its A3 domain with matrix collagen. This induces a conformational change in the A1 domain, thereby allowing interaction with glycoprotein (GP) platelet receptor Ib-IX-V. This interaction stimulates calcium release, subsequent platelet activation, and subsequent conformational change of the fibrinogen receptor (GPIIb/IIIa), which can interact with fibrinogen and vWf to favor the interaction between platelets (platelet aggregation process). ADP indicates adenosine diphosphate; RGD and Arg-Gly-Asp are amino acid sequences (adapted from Badimon et al., 2009 [30]).

**Figure 7:**
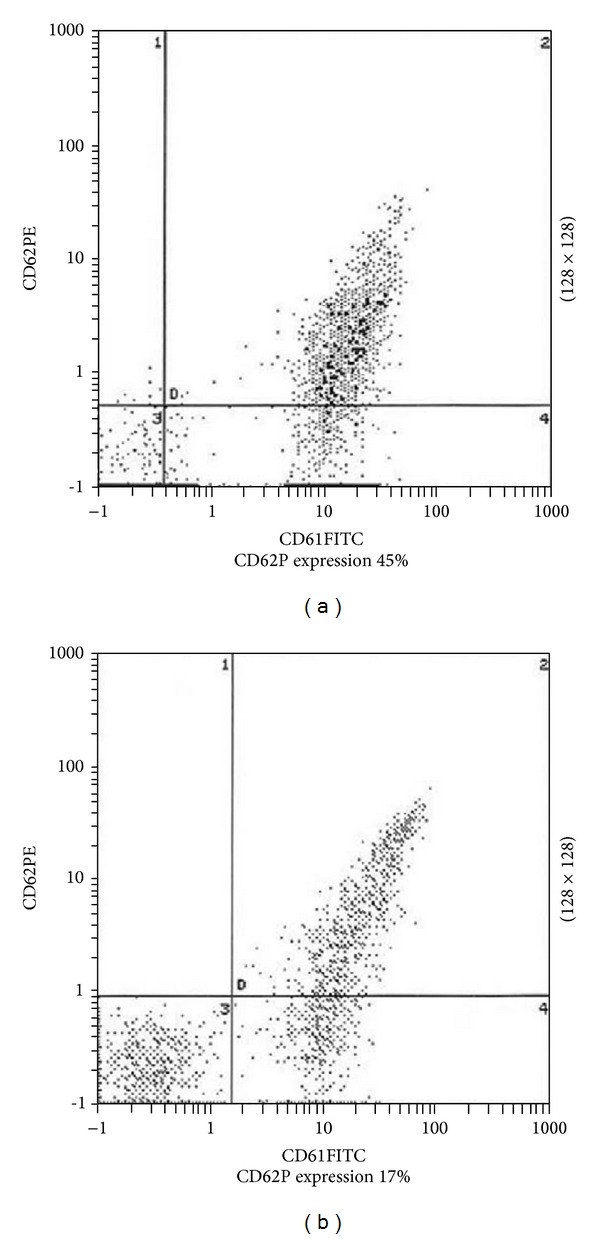
Flow cytometric detection of P-selectin in (a) diabetic patient and (b) nondiabetic patient (adapted from Saad et al., 2011 [34]).

**Figure 8:**
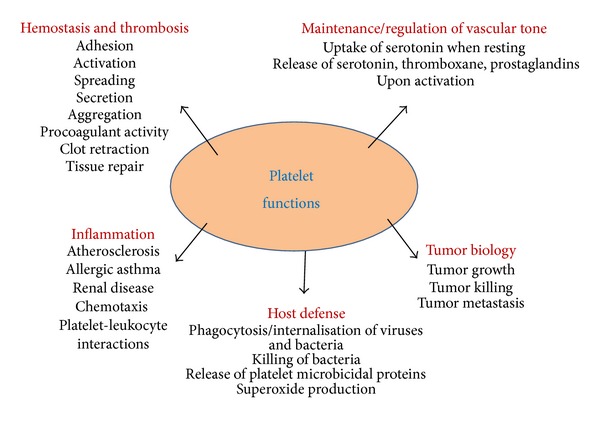
The multifunctional platelet (adapted from Harrison, 2005 [44]).

One of the most common changes in platelet behaviour in diseased condition, such as diabetes, is platelet hyperaggregation. In response to various agonists, hyperaggregation in platelets was reported in patients with both type 2 and type 1 diabetes mellitus [55, 56]. Hyperaggregation is associated with generating more 11-dehydro-thromboxane B_2_ which is the important end product of thromboxane pathway as discussed earlier (Figure 2). DM has been reported to increase the production of this product as well as prothrombin accelerating aggregation; this can increase the chances of CVD among diabetic patients, as hyperaggregated platelets have a tendency to block the blood vessels. By contrast, anticoagulant markers, such as activated protein C, protein C activation peptide, and soluble thrombomodulin (TM), were depressed in T2DM, further increasing the chances of CVD [57].

### 4.2. Alterations in Thromboxane Production

Thromboxane is an important product which plays profound role in platelet aggregation. Studies showed that thromboxane production is enhanced in diabetes subjects compared to controls increasing platelet aggregation, indicating a higher risk of CVD [58–60]. To initiate aggregation, platelets from patients with T2DM can synthesize greater amount of TxB_2_ but it requires less arachidonate, the precursor of thromboxane pathway and collagen, than from normal nondiabetic controls [61]. TxA_2_ production has been positively correlated with fasting plasma glucose and HbA1c; higher blood glucose increases the production of TxA_2_ which is an important product in thromboxane pathway. Several studies showed reduced TxA_2_ production in improved glycaemic controls [58, 62, 63]. Data suggests that 8-iso-PGF2*α*, a marker involved in lipid and arachidonate peroxidation; is correlated with TxA_2_ biosynthesis which could be a link between glycaemic control, oxidative stress, and platelet activation [64]. Thus alteration in thromboxane pathway can be a link between platelet dysfunction and diabetes, obesity, and CVD.

### 4.3. Changes in Platelet Membrane Fluidity

Change in membrane fluidity has been assigned to the impairment of platelet functions since membrane fluidity can modulate cell function, and reduced membrane fluidity in cholesterol- enriched platelets is associated with platelet hypersensitivity to different agonists [65]. Due to glycation of membrane proteins, platelet membrane fluidity has been grossly impaired in diabetes. Decreased membrane fluidity of these platelets is attributed to an increased cholesterol-phospholipid molar ratio in platelet membranes. Reduced membrane fluidity is associated with hypersensitivity to thrombin in intact platelets from diabetic subjects [66, 67].

### 4.4. Altered Expression of Platelet Agonists

The number and adhesiveness of several platelet specific surface glycoprotein receptors (GP) are significantly enhanced in diabetic subjects. CD40 ligand on platelets has been correlated with HbA1c concentrations. An upregulation of the CD40-CD40 ligand system has been observed in patients with DM [68] along with an increase in GPIIb/IIIa, vWf, GPIa/IIa, P-selectin (CD62), and CD63 [69–72]. Patients suffering from ischemic heart disease (IHD) and depression concurrently may have abnormal platelet activation resulting in an increased risk of thrombosis. Mean PF4 and **β**-TG plasma levels in the IHD group with depression were found to be significantly higher than those of the control and IHD groups [73]. Moreover high vWf value may increase the chance of CVD [74] which can also act as an index for disease prediction.

Hyperglycemia induces downregulation of SIRT1 and upregulation of PAF-R in endothelial progenitor cells whose association is linked with vascular complications [75]. Platelet activity is correlated with nitric oxide (NO) synthesis, which is impaired in acute coronary syndrome (ACS). Significant differences of eNOS gene polymorphism and expression have been observed between diabetic patients with ACS and patients with ACS but no history of diabetes [76]. Platelet FcgammaRIIA is significantly overexpressed in type 2 diabetes whose interaction with collagen may alter platelet adhesion.

Several candidate gene polymorphisms have been associated with platelet functions. Polymorphisms of these genes may develop CAD, CVD, and ACS in both diabetic and nondiabetic subjects. Some of these genes are glycoprotein Ia/IIa (*α* ^2^ *β* ^1^) (—807C>T, 873G>A—, —837C>T, 1648A>G), glycoprotein lb*α* (VNTR A-D), and glycoprotein IIb/IIIa (substitution of proline for leucine at position 33) [77]. However, alpha 2 integrin C807T gene polymorphism plays a crucial role in arterial thrombosis [78].

### 4.5. Changes in Mean Platelet Volume (MPV)

MPV is machine generated average size of a platelet, whose value is enhanced in subjects with DM. An explanation of this phenomenon is that larger platelets are more reactive and can express more surface receptors [88, 89]. Increased MPV value is associated with diabetic retinopathy. Event of myocardial infarction is positively correlated with MPV [90].

### 4.6. Alterations in Intracellular Ionic Environment

Platelet hyperactivity is associated with reduced Na^+^/K^+^ ATPase and increased Ca^2+^ ATPase activity which can increase intracellular Ca^2+^ concentration, thus increasing platelet activity [91]. Moreover a decrease in intracellular Mg^+^ concentration in diabetic platelets may provoke an accelerating effect in platelet activity [92]. Oxidative stress is associated with superoxide anion production and nitric oxide (NO) synthesis as well as production of reduced glutathione. Hyperglycemia exerts oxidative stress which can further implement altered platelet functions [93].

### 4.7. Changes in Mitochondrial Membrane Potential

Studies have established that plasma glycated haemoglobin (HbA1c) level is positively correlated with platelet ATP content, suggesting that hyperglycemia promotes platelet mitochondria to generate more ATP, but decreases platelet mitochondrial potential [94].

### 4.8. Effect of Antidiabetic Drugs on Platelet Dysfunction

Apart from aspirin which is commonly used in diabetes mellitus, many widely used antidiabetic drugs also result in platelet malfunction. The majority of these drugs are presented hereunder.

#### 4.8.1. Metformin

Metformin, an oral antidiabetic, first line drug of biguanide class, is particularly useful for overweight and obese diabetic patients. Chakraborty et al. reported that metformin is particularly useful to restore the antioxidant status of cells hampered in type 2 diabetes [95]. Metformin treatment also decreases ROS generation and creates mitochondrial membrane hyperpolarization lowering the risk of CVD [96]. Different researchers concluded that metformin can stabilize platelet by reducing platelet density, *β*-TG, and platelet superoxide anion production [97–99]. Metformin also helps to reduce PAI-1 in plasma, thus putting beneficial effects on fibrinolysis. By reducing FVII and FXIII, metformin also plays an important role in coagulation [100].

#### 4.8.2. Sulfonylurea

Sulfonylurea derivatives are a class of antidiabetic drugs which act by increasing insulin release from the beta cells in the pancreas. *Gliclazide*, a sulfonylurea derivative, can check blood glucose level by its free radical scavenging ability. It also confers beneficial effects on the hemorrheologic abnormalities by reducing platelet abnormalities in diabetic patients by increasing prostacyclin synthesis [101]. Gliclazide can reduce platelet aggregation, enhance fibrinolysis, inhibit the surface expression of endothelial adhesion molecules, and inhibit neutrophil endothelial cell adhesion [102, 103].

#### 4.8.3. Thiazolidinediones

Thiazolidinediones or glitazones activate PPAR*γ* via FFA and eicosanoids. When activated, the receptor binds to DNA in complex with the retinoid X receptor (RXR) modifying the transcription of certain genes in several tissues including vascular tissue [104]. Platelets have profound role in inflammation due to their release of the proinflammatory and proatherogenic mediators: CD40 ligand (CD40L) and thromboxanes (TXs). Studies showed that platelet incubation with rosiglitazone prevented thrombin-induced CD40L surface expression and release of CD40L and thromboxane B_2_(TxB_2_) [105]. Administration of rosiglitazone has significantly reduced von Willebrand factor in one study [106]. Platelets from patients with type 2 diabetes mellitus lack platelet endothelial cell adhesion molecule-1. Rosiglitazone treatment has been found to increase platelet SERCA-2 expression and Ca^2+^-ATPase activity. Moreover, it has inhibiting effect on SERCA-2 tyrosine nitration, thus normalizing platelet Ca^2+^ concentration. Rosiglitazone also reduces *μ*-calpain activity, normalizes platelet endothelial cell adhesion molecule-1 levels, and partially restores platelet sensitivity to nitric oxide synthase inhibition [107].

#### 4.8.4. Acarbose

Acarbose blocks carbohydrate digestion by inhibiting enzyme glycoside hydrolases. Acarbose is known to decrease platelet-monocyte aggregate formation by reducing postprandial hyperglycaemia in diabetic patients [108].

### 4.9. Effects of Insulin on Platelets

Insulin resistance and associated metabolic syndrome jointly correlate with prothrombic state in diabetes mellitus to put a cumulative effect in the progression of vascular complications [109]. Insulin has been reported to have antiplatelet effects. Researchers have identified a direct correlation between insulin-induced attenuation of the thrombin-induced Ca^2+^ response and platelet aggregation [110, 111]. Diabetic platelet shows an altered Ca^2+^ homeostasis [112]. Tyrosine phosphorylation of platelet plasma membrane Ca^2+^-ATPase may serve as positive feedback to inhibit membrane Ca^2+^-ATPase and increase intracellular calcium during platelet activation [113].

Reports suggest that in nondiabetic patients with acute ischemic heart disease binding of PGI_2_ with insulin is lessened [114]. But insulin administration has normalized platelet activity to PGI_2_ in coronary heart diseases both *in vitro* and *in vivo* [115, 116]. Vitamin E deficiency plays a crucial role in platelet aggregation, where *α*-tocopherol supplementation in response to ADP decreases TxA_2_ [117]. Although several studies concluded the beneficial role of Vitamin E supplementation in diabetic patients with CVD, large scale study fails to conclude the minimization of myocardial infarction occurrence in diabetic patients [118]. Insulin therapy may improve platelet sensitivity to NO, thus providing a suitable platform in treating CVDs among diabetic patients.

## 5. Platelet Dysfunction in Other Diseases

### 5.1. Heart Disease

Not only diabetes related cardiovascular disorder but also platelet dysfunction has been reported in other heart diseases. It has been reported in many cases that patients with congestive heart failure (CHF) have increased risk of venous thromboembolism, stroke, and sudden death [119]. J. Mehta and P. Mehta first reported that patients with CHF have significantly higher number of circulating platelet aggregates than the normal subjects [120]. In one study, patients with left ventricular dysfunction have shown increased number of fibrin D-dimer, fibrinogen and vWf levels compared to healthy controls [121]. Patients with acute decompensated heart failure (AHF) show more abnormalities of platelet activation than stable CHF patients and healthy controls [122]. In heart failure, endothelial nitric oxide (NO) production is much lower where oxidative stress and NO degradation rate are much higher. Platelet has been shown to produce less bioactive NO in patients with heart failure, mainly due to the defect in the the platelet l-arginine/NO/guanylyl cyclase pathway [123]. Thus altered platelet activity can give major insights into heart disease and can be an interesting field to explore.

### 5.2. Renal Disease

Complex hemostatic disorders have been found in patients with end stage renal disease (ESRD) that may be in form of bleeding diatheses. Platelet dysfunctions result due to the presence of toxic products in the circulating blood. Dialysis improves this complication; however, it does not eliminate the risk of hemorrhage. Some common pathological features include thrombocytopenia, glomerular thrombosis, and thrombi in small arteries and glomerular capillaries [124]. Both intrinsic platelet abnormalities and impaired platelet-vessel wall interaction can contribute to platelet dysfunction [125]. In renal failure, the normal activation process of the platelets to form aggregates is impaired.

### 5.3. Cancer and Tumorigenesis

Platelet plays versatile role in cancer progression. The procoagulant environment provided by platelets can secure the coagulation of cancer cells, protecting them from immune system, thus prompting the formation of tumors [126]. Platelets facilitate tumor cell migration and invasiveness, prompting metastasis. It has been reported in both breast and ovarian cancer that platelets increase the invasiveness of cancer cells which can induce the further progression of the disease [127–129]. Moreover, tumor cells also have the ability to aggregate platelets [130], further increasing the chance of inducing metastasis. Activation of platelets and regulation of other cells have been controlled by thrombin by means of G protein-coupled protease-activated receptors (PARs) [131]. Researchers have shown that thrombin signaling also contributes hugely to the progression of tumorigenesis and angiogenesis [132].

### 5.4. Alzheimer's Disease

Platelet dysfunction has been also implicated in Alzheimer's disease (AD), which is the most common form of dementia. Platelet can store a huge amount of amyloid precursor protein (APP**)**; recent finding shows that platelet APP metabolism may accumulate A*β* in the brain and its vasculature through the blood brain barrier [133]. Platelet *α*-granules store RANTES, an inflammatory signaling molecule whose secretion from PBMC has been reported to increase in AD [134]. The fluidity of the hydrocarbon region of platelet membranes from the AD patients is significantly higher than that of control group [135]. Platelet secretases activities and COX enzyme activity (which is also a component in APP secretion pathway) have been impaired in platelets of AD patients, thus making this blood particle a suitable marker of this disease [136].

### 5.5. Liver Disease

Quantitative and qualitative platelet defects, hyperfibrinolysis, accelerated intravascular coagulation, and decreased synthesis of clotting and inhibitor factors are well observed in both acute and chronic liver diseases [137]. As most coagulation factors except vWf are synthesized by liver parenchymal cells, and liver's reticuloendothelial system plays crucial role in the clearance of activation products, a defect in liver function may alter hemostasis [138, 139]. Not only functions but platelet numbers also have been reported to decrease in liver diseases [140]. It has been reported that platelet transfusion can improve liver function in patients with chronic liver disease and cirrhosis [141]. Platelets morphological parameters have been also reported to alter in liver diseases [142], thus providing a platform for platelet research in liver diseases.

## 6. Conclusion

Biomarkers are now emerging with immense scientific and clinical value through the whole journey of the disease process. Before diagnosis, markers can be used for prediction, screening, and risk assessment. During diagnosis, markers can determine staging, grading, and selection of initial drug therapy. During treatment, markers are used to monitor the prognosis of therapy or to select additional treatment needed. Platelet indices indicating differential dysfunction, hyperactivation, aggregation, or adhesion are capable of expressing the characters of disease pathogenesis. Accurate determination of platelet indices is cost effective and their impaired function can be correlated with the inflammation involved in diseased state. Thus, an attempt to identify different platelet biomarkers is of significant impact on the global scene of clinical application and development.

## Figures and Tables

**Table:** (a) Platelet receptors in recruitment, adhesion, and aggregation

Receptors	Present in	Family	Ligands	Comments
Initiation of platelet recruitment				
GPIb-IX-V complex	Platelet surface	Leucine-rich repeat family	vWf, thrombin, FXI, FXII, P-selectin, HK, Mac-1, TSP-1	Bernard-Soulier syndrome

Platelet adhesion and aggregation				
GPVI	Platelet surface	Ig superfamily	Collagen, laminin	
*α*_2_ *β*_1_	Platelet plasma membrane	Integrins	Collagen	
*α*_5_ *β*_1_	”	”	Fibronectin	
*α*_6_ *β*_1_	”	”	Laminin	
*α*V*β*_3_	”	”	Vitronectin, fibrinogen, vWf, osteopontin	
*α*_IIb_ *β*_3_	”	”	Fibrinogen, fibrin, vWf, TSP-1, fibronectin, vitronectin	Glanzmann thrombasthenia
CD148	Platelet surface	Tyrosine phosphatase receptor	Unknown	Regulation of GPVI
CLEC-2	”	C-type lectin receptor	Podoplanin (platelets? CLEC-2?)	

**Table:** (b) Platelet receptors in the amplification phase

Receptors	Present in	Family	Ligands	Comments
P2Y_1_	Platelet plasma membrane	G protein-coupled receptors	ADP	
P2Y_12_	”	”	”	
PAR1	”	”	Thrombin	High affinity
PAR4	”	”	”	Low affinity
tPA	”	”	Thromboxane	
PAF receptors	”	”	1-O-alkyl-2-acetyl-sn-glycero-3-phosphocholine	PAF: platelet activating factor
PGE_2_ receptor (EP_3_)	”	”	PGE_2_	
Lysophosphatidic acid receptor	”	”	Lysophosphatidic acid	
Chemokine receptors	”	”	Chemokines	
V1a vasopressin receptor	”	”	Vasopressin	
A2a adenosine receptor	”	”	Adenosine	
b2 adrenergic receptor	”	”	Epinephrine	
Serotonin receptor	”	”	Serotonin (5-hydroxytryptamin)	
Dopamine receptor	”	”	Dopamine	
P_2_X_1_	”	Ion channel	ATP	
c-Mp1	”	Tyrosine kinase receptor	TPO	
Insulin receptor	”	”	Insulin	
PDGF receptor	”	”	PDGF	
Leptin receptor	”	Cytokine	Leptin	

**Table:** (c) Platelet receptors in the stabilization phase and in the negative regulation of platelet activation

Receptors	Present in	Family	Ligands	Comments
Stabilization				
Eph receptor	Platelet plasma membrane	Tyrosine kinase receptor	Ephrin	
Axl/Tyro3/Mer	”	”	Gas-6	
P-selectin	Platelet *α* granule; comes in plasma membrane upon activation	C-type lectin receptor family	PSGL-1, GPIb, TF	Soluble P-selectin: biomarker
TSSC6	Platelet plasma membrane	Tetraspanins	—	
CD151	”	”	—	
CD36	”	Class B scavenger receptor	TSP1, oxLDL, VLDL, oxPL, collagen type V	Many functions
TLT-1	”	Ig superfamily	Fibrinogen?	TLT-1 soluble form correlated with DIC
PEAR1	”	Multiple EGF-like domain protein	—	Phosphorylated after platelet contact

Negative regulation				
VPAC1	Platelet plasma membrane	G protein-coupled receptors	PACAP	
PECAM-1	”	Ig superfamily	PECAM-1, collagen, glycosaminoglycans	
G6B-b	”	”	—	
PGI_2_ receptor (IP)	”	G protein-coupled receptors	PGI_2_	Prostacyclin released from endothelial cells
PGD_2_ receptor	”	”	PGD_2_	
PGE_2_ receptor (EP4)	”	”	PGE_2_	

**Table 2:** A comparison of endothelial and platelet properties (adapted from Warkentin et al., 2003 [21]).

	Endothelium	Platelets
Nucleus	Yes	No
mRNA	Lots	Little but active
Cell dimensions	Highly variable: up to 100 *μ*M length in large vessels	Diameter ~4 *μ*M, volume 7–12 fL (inversely proportional to platelet count)
Life span	Long (months to years)	Short (7–9 days)
Daily production	Not known	2.5 × 10^11^
Circulating	Few	Most (normally 1/3 sequestered in spleen; may become sequestered on activated endothelium)
Diagnostic markers	Indirect and not clinically useful	CBC, peripheral smear, platelet function studies
Origin	Bone marrow	Bone marrow
Storage granules	Weibel-Palade bodies	*α* granules, dense granules, lysosomes
Partial list of storage components	vWf, P-selectin, multimerin	vWf, P-selectin, multimerin, fibrinogen, PDGF, TGF-*β*, IL-1, VEGF, angiopoietin, RANTES, PF4, ADP, ATP, serotonin

**Table 3:** Clinical assessment of platelet functions.

Platelet aggregometry	
Photo-optical platelet aggregometry [79]	Platelet-rich plasma is taken to check the platelet aggregation.
Impedance platelet aggregometry [80]	Platelet aggregation is checked by using the whole blood by electrical impedance.
Light-scattering platelet aggregometry [81]	It is a combination of laser light scattering and aggregometry to monitor platelet microaggregate formation.

Point-of-care for platelet function tests	

Ultegra rapid platelet function assay (RPFA) [82]	RPFA is a simple and fast, automated point-of-care device that monitors GPIIb-IIIa inhibition. This test is based on platelet agglutination from interaction between unblocked GPIIb-IIIa receptors and fibrinogen-coated beads.
Platelet-activating clotting test (PACT) assay [83]	The PACT assay, HemoSTATUS, measures ACT without a platelet activator, comparing it with ACTs obtained with increasing concentrations of platelet-activating factor (PAF).
Platelet function analyzer (PFA-100) [84]	PFA-100 exposes platelets within citrated whole blood to high shear stress through a capillary tube, followed by an aperture in a membrane coated with collagen and either ADP or epinephrine. The platelets adhere and aggregate until the aperture is occluded, and the time to this closure is recorded.
Plateletworks test [85]	The Plateletworks is a point-of-care test that uses a Coulter counter to measure platelet-count ratio, red blood cell count, hemoglobin, and hematocrit. Platelet count is measured in a control sample in which aggregation is prevented by EDTA and compared with the platelet count in an agonist-stimulated (ADP or collagen) sample.
Clot signature analyzer [86]	This test uses nonanticoagulated whole blood and can measure several aspects of platelet function and clotting properties.
Thromboelastography [87]	This device measures clot strength and gives a global assessment of hemostasis.
